# Genome-Wide Identification and Expression Analysis of WRKY Gene Family in *Neolamarckia cadamba*

**DOI:** 10.3390/ijms24087537

**Published:** 2023-04-19

**Authors:** Zuowei Xu, Yutong Liu, Huiting Fang, Yanqiong Wen, Ying Wang, Jianxia Zhang, Changcao Peng, Jianmei Long

**Affiliations:** 1 Guangdong Key Laboratory for Innovative Development and Utilization of Forest Plant Germplasm, College of Forestry and Landscape Architecture, South China Agricultural University, Guangzhou 510642, China; scau-zuowei@stu.scau.edu.cn (Z.X.); lyt991211@stu.scau.edu.cn (Y.L.); 18125965000@163.com (H.F.); wenwen_yq@163.com (Y.W.); wang_scau_wind@163.com (Y.W.); zhangjianxia@scau.edu.cn (J.Z.); 2 State Key Laboratory for Conservation and Utilization of Subtropical Agro-Bioresources, South China Agricultural University, Guangzhou 510642, China

**Keywords:** *WRKY* gene, *Neolamarckia cadamba*, abiotic stress, expression analysis, cadambine

## Abstract

The WRKY transcription factor family plays important regulatory roles in multiple biological processes in higher plants. They have been identified and functionally characterized in a number of plant species, but very little is known in *Neolamarckia cadamba*, a ‘miracle tree’ for its fast growth and potential medicinal resource in Southeast Asia. In this study, a total of 85 *WRKY* genes were identified in the genome of *N. cadamba*. They were divided into three groups according to their phylogenetic features, with the support of the characteristics of gene structures and conserved motifs of protein. The *NcWRKY* genes were unevenly distributed on 22 chromosomes, and there were two pairs of segmentally duplicated events. In addition, a number of putative cis-elements were identified in the promoter regions, of which hormone- and stress-related elements were shared in many *NcWRKYs*. The transcript levels of *NcWRKY* were analyzed using the RNA-seq data, revealing distinct expression patterns in various tissues and at different stages of vascular development. Furthermore, 16 and 12 *NcWRKY* genes were confirmed to respond to various hormone treatments and two different abiotic stress treatments, respectively. Moreover, the content of cadambine, the active metabolite used for the various pharmacological activities found in *N. cadamba,* significantly increased after Methyl jasmonate treatment. In addition, expression of *NcWRKY64/74* was obviously upregulated, suggesting that they may have a potential function of regulating the biosynthesis of cadambine in response to MeJA. Taken together, this study provides clues into the regulatory roles of the *WRKY* gene family in *N. cadamba*.

## 1. Introduction

The WRKY proteins are one of the largest families of transcriptional regulators found throughout plants [1]. They share the defining feature WRKY domain which comprises the highly conserved WRKYGQK hepta peptide sequence at the N-terminal followed by a C_2_H_2_- or C_2_HC type of zinc-finger motif at the C-terminal. Both the WRKY domain and zinc-finger motif are required for the high binding affinity of WRKY transcription factor (TF) to the W-box cis-elements in the promoter regions of their target genes [2]. The WRKY proteins can be classified into three main groups (I–III) based on the number of WRKY domains and the category of zinc-finger motifs. Proteins with two WRKY domains belong to Group I, while Group II or III contain one WRKY domain with different zinc-finger motifs. Specifically, Group III has a zinc-finger structure of C_2_HC while Group II has a zinc-finger structure of C_2_H_2_ and can be further divided into five subgroups: IIa, IIb, IIc, IId, and IIe [3]. 

Many WRKY TFs have been experimentally identified from various plant species. They play vital regulatory roles in plant defense regulatory networks, including response to various abiotic stresses, which result from an interplay between WRKYs and a variety of plant hormones [4,5]. For instance, the synergistic interaction between OsWRKY51 and OsWRKY71 genes inhibited gibberellic acid (GA) signaling in the aleurone cells of rice seeds under ABA induction in rice [6,7]. Likewise, in Arabidopsis, AtWRKY18/40/60 were shown to participate in signaling pathways that are mediated by ABA, and *AtWRKY60* might be a direct target gene of AtWRKY18 and AtWRKY40 in ABA signaling [8]. Moreover, *PoWRKY13* in *Populus tomentosa* [9], *PheWRKY86* in *Moso bamboo* [10] and *AtWRKY25/26/39* [11,12] were involved in the response to heat stress and drought stress. *DgWRKY5* in *Chrysanthemum indicum* [13], *GbWRKY1* in cotton [14], and *SlWRKY8* in *Solanum lycopersicum* [15] participated in regulating plant tolerance to salt stress. 

WRKY TF also plays an activating or repressing role in the transcriptional regulation of key enzyme genes in plant secondary metabolites synthesis [4]. OpWRKY2 acted as a direct positive regulator of camptothecin biosynthesis by binding the central camptothecin pathway gene OpTDC [16]. Similarly, the total production of camptothecin was significantly upregulated in most overexpression lines of *OpWRKY3* [17]. Overexpression of *OpWRKY6* significantly reduced the accumulation of camptothecin. Conversely, camptothecin accumulation increased in *OpWRKY6* knockout lines [18]. Agarwood sesquiterpene synthase 1 (ASS1) is one of the key enzymes responsible for the biosynthesis of sesquiterpenes, and *AsWRKY44* directly binds to its promoter and represses ASS1 promoter activity [19]. Likewise, *GaWRKY1* and *AaWRKY1* activated the expression of key enzyme genes in the gossypol and artemisinin biosynthesis pathway, respectively, by binding to the W-box element in their promoter [20,21]; *PgWRKY4X* in ginseng interacted with the W-box in the squalene epoxidase (PgSE) promoter and overexpression of *PgWRKY4X* significantly upregulated PgSE and increased the accumulation of ginsenoside [22]. Paclitaxel, as a kind of terpenoid alkaloid, also has important clinical value in the treatment of cancer. Overexpression of *TcWRKY8* and *TcWRKY47* significantly increased the expression levels of genes related to paclitaxel biosynthesis [23]. Extracted from *Catharanthus roseus*, vinblastine is a monoterpenoid indole alkaloid (MIA) whose synthetic pathway has been fully resolved and is a natural antitumor drug widely used in cancer therapy. Overexpression of *CrWRKY1* in *C. roseus* hairy root activated some key genes in the MIA pathway and the transcriptional repressors (*ZCT1*, *ZCT2*, and *ZCT3)*. Interestingly, CrWRKY1 overexpression repressed the transcriptional activators *ORCA2*, *ORCA3*, and *CrMYC2*, yielding a higher level of serpentine accumulation [24]. To date, however, the regulation of WRKY in MIA synthesis is less understood. 

*Neolamarckia cadamba*, from the *Rubiaceae* family, is widely distributed in tropical and subtropical regions of the world and is an important tree for the timber industry and traditional medicinal plants in southern China [25]. Cadambine, a kind of MIA only isolated from the *Rubiaceae* family, is the main component of total alkaloids in *N. cadamba*, accounting for about 50% of total alkalis, which has been shown to have clinical effects against malaria parasites and in the treatment of diabetes, and exhibits concentration-dependent inhibition on carcinoma cell and DNA topoisomerase [26,27,28]. With the completion of whole genome sequencing of *N. cadamba* [29], it enabled genome-wide identification and functional analysis of the gene families related to the development process, response to environmental change, and cadambine synthesis. Several reports suggested that WRKY participated in the regulation of MIA biosynthesis, but whether they were involved in cadambine biosynthesis was unknown. In this study, we identified 85 *WRKY* genes of *N. cadamba* at the genome-wide level and analyzed the gene structures, conserved domains, phylogenetic relationships and cis-element in the promoter of the *WRKY* genes. In addition, we performed a comprehensive analysis of spatiotemporal expression patterns of *NcWRKY* genes according to RNA-seq data and examined their expression profiles in response to hormone (MeJA, ABA, and GA) and abiotic stresses (high salinity and drought) by real time quantitative PCR (RT-qPCR). Moreover, the involvement of the NcWRKY in cadambine synthesis was further explored. Our genome-wide results identified all *WRKY* genes in the *N. cadamba* genome and provided valuable clues for further functional study. 

## 2. Results

### 2.1. Identification of WRKY Genes in Neolamarckia Cadamba

In this study, a total of 85 *WRKY* gene sequences were identified in *N. cadamba* (Supplementary File S1). They were named based on the apparently complete WRKY domains and their position on the chromosome. Gene characteristics, including the length of the protein sequence, the protein molecular weight (MW), the isoelectric point (pI), and the subcellular localization, were analyzed using ExPaSy and Plant-mPLoc. The results showed that the length of all the identified WRKY proteins in *N. cadamba* ranged from 174 to 734 amino acids (aa), in which *NcWRKY48* was the smallest protein and *NcWRKY47* was the largest one. The molecular weight (MW) of the NcWRKY proteins ranged from 20.0 to 79.3 kDa, with the predicted isoelectric point values (pI) varying from 5.04 (NcWRKY6) to 9.69 (NcWRKY61). Subcellular localization prediction indicated that all the NcWRKYs were localized to nuclear, indicating that they may function as transcription factors.

### 2.2. Phylogenetic Analysis and Multiple Sequence Alignment of NcWRKYs

**Figure 1:**
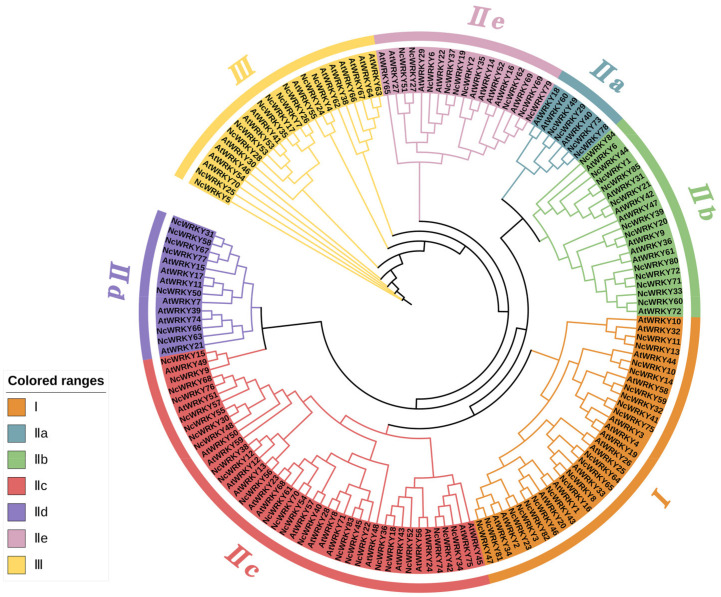
Unrooted phylogenetic tree representing relationships among WRKY proteins of *N. cadamba* and *A. thaliana*. A maximum likelihood (ML) phylogenetic tree was constructed with 5000 bootstrap replicates. The different-colored arcs indicate different groups (or subgroups) of WRKY proteins. WRKY proteins from *N. cadamba* and *A. thaliana* were presented with the prefix ‘Nc’ and ‘At’, respectively.

To investigate the phylogenetic relationships among WRKY family genes, we constructed an ML phylogenetic tree using the WRKY proteins from *A. thaliana* and *N. cadamba*. The result showed that the 85 NcWRKY proteins were classified into three groups (Figure 1), based on the classification described by a previous report on *A. thaliana* [3], indicating an evolutionary conservation between these two species. Group I contained 19 NcWRKY members, while 10 NcWRKYs belonged to Group Ⅲ. The largest group (Group II) consists of 56 NcWRKY proteins, which could be further divided into five subgroups, with 4, 12, 24, 7, and 9 members belonging to Group IIa–IIe, respectively. Multiple sequence alignment analysis (Figure S1) suggested that members from Group I contained two WRKY domains and C_2_H_2_-type zinc-finger motifs (C-X_4_-C-X_22–23_-H-X-H), except that NcWRKY23 only harbored two WRKY domains. All the 56 NcWRKYs from Group Ⅱ contained one WRKY domain, of which six harbored mutated WRKY domains (WRKYGKK). All members in Group III contained the C_2_HC-type zinc fingers (C-X_7_−C-X_23_-H-X-C).

### 2.3. Gene Structure and Conserved Motif Analysis

**Figure 2:**
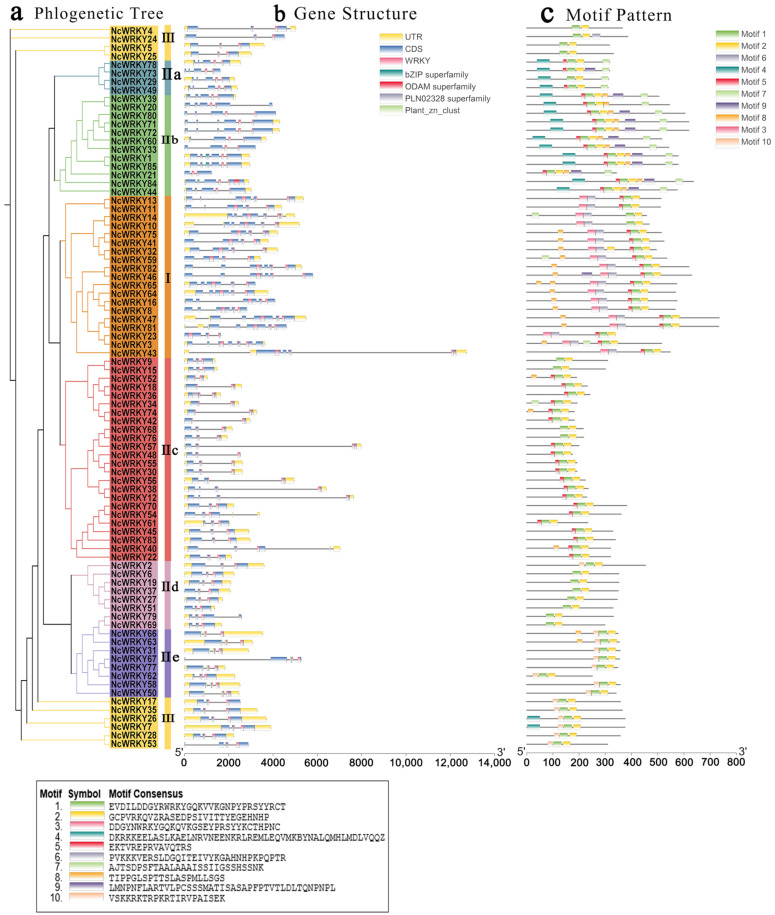
Phylogenetic relationships, gene structure, and architecture of conserved protein motifs in WRKY genes from *N. cadamba*. (**a**) The phylogenetic tree was constructed based on the full-length sequences of NcWRKY proteins using TBtools. Details of clusters were shown in different colors as same of Figure 1. (**b**) Exon–intron structure of NcWRKYs. Yellow boxes indicated untranslated 5′- and 3′ regions, while blue boxes indicated coding sequence (CDS) and black lines represented introns. The WRKY domains were highlighted by pink boxes and other colors indicate different conserved domains which are found in CDD. The length of genes was estimated using the scale at the bottom. (**c**) The motif composition of NcWRKY proteins. The motifs, numbers 1–10, were displayed in different-colored boxes.

The exon–intron organizations of all the identified NcWRKYs were examined by TBtools. Another ML phylogenetic tree was constructed to align the 85 NcWRKY proteins (Figure 2a). As shown in Figure 2b, the number of introns in the *NcWRKYs* ranged from 1 to 6. Six *NcWRKY* genes from Group Ⅱc had only one intron, and NcWRKY3 had six introns. The majority of *NcWRKY* genes contained two to five introns (37 with two introns, 13 with three introns, 17 with four introns, and 11 with five introns). Interestingly, all *NcWRKYs* in Group I contained an intron in their respective WRKY domains, but no introns existed in the N-terminal WRKY domains (Figure 2b). Genes within the same group were usually similar in structure, slightly varying in length and distribution, and most genes had UTR regions. For example, all of Group IIa genes had four introns, and there were two UTR regions in all of them except for *NcWRKY73*. Most of Group IIc contained one to three introns, but *NcWRKY40* and *NcWRKY54* contained four introns and one of them was located in 3′ UTR.

To further investigate the similarity and diversity of the motif composition of NcWRKY proteins, we annotated 10 conserved motifs predicted by MEME. As exhibited, NcWRKY members in the same subfamily were found to share similar motif profiles (Figure 2c). Motif 1 and motif 2 were highly conserved and distributed across all members of NcWRKY. Most members in Group I and Group IIb contained the highest number of motifs (n = 7), whereas three members in Group Ⅲ (NcWRKY4, NcWRKY5, and NcWRKY25) only have two motifs. In addition, we also found that some motifs were specific to different groups. For example, motif 3 and motif 6 were unique to Group I, whereas motif 9 was specific to Group IIa and IIb. The distinct motif composition might be contributed to the functional diversity among NcWRKYs. 

### 2.4. Chromosomal Distribution and Synteny Analysis of NcWRKYs

**Figure 3:**
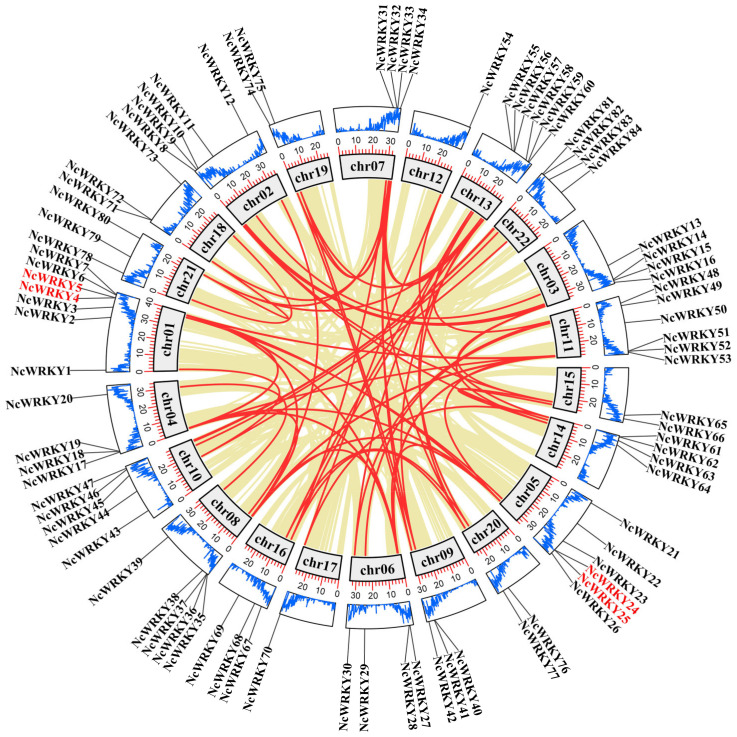
Chromosome location and synteny analysis of *WRKY* genes within *N. cadamba* genome. Khaki lines indicated all synteny blocks in the *N. cadamba* genome, and the red lines indicated duplicated *WRKY* gene pairs. The gray boxes indicated the chromosomes of *N. cadamba*. The blue line in the boxes represented the gene abundance at that position on the chromosome, and the height of the line was proportional to the abundance. The outermost circle indicates the chromosomal locations of *WRKY* genes in *N. cadamba*. The genes in red font indicate tandem duplication.

An analysis of genome chromosomal distribution revealed that 84 *NcWRKYs* were unevenly distributed on 22 chromosomes (Figure 3), while *NcWRKY85* could not map to any chromosome. There was no evidence to show a correlation relationship between the number of *NcWRKYs* and the chromosome length. The investigation of gene duplication events was also performed to obtain insight into the expansion of the *NcWRKY* family. Four *NcWRKYs* (*NcWRKY4/5* and *NcWRKY24/25*) were identified as tandem repeat gene pairs located on chr01 and chr05. In addition to tandem duplication, the fragment duplication events of the *WRKY* gene family were performed (Figure 3). The result showed that 76 segmental duplication events with 62 *NcWRKYs* were identified. All these results indicated that the *NcWRKY* family underwent an expansion in *N. cadamba* possibly generated by gene duplication, and the segmental duplication events played a major role as a driving force for *NcWRKYs* evolution. To better understand the evolutionary constraints acting on the *NcWRKY* family, the Ka/Ks ratios of the *NcWRKY* gene pairs were calculated. All segmental and tandem duplicated *NcWRKY* gene pairs had Ka/Ks < 1 (Supplementary File S2), suggesting that the *N. cadamba WRKY* gene family might have experienced strong purifying selective pressure during evolution.

**Figure 4:**
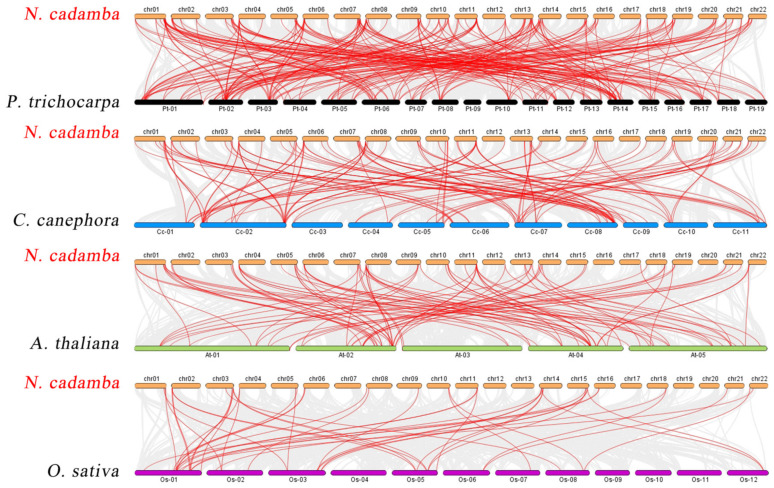
Synteny analysis of *WRKY* genes between *N. cadamba* and four representative plant species. Gray lines in the background indicated all synteny blocks in the genome, while the red lines indicated the duplication of *WRKY* gene pairs.

The phylogenetic mechanisms of the *N. cadamba* WRKY family were further explored by constructing comparative syntenic maps of *N. cadamba* associated with four representative species, including three represented dicots (*A. thaliana*, *Coffea canephora*, and *Populus trichocarpa*) and one model monocot (*O. sativa*) (Figure 4). On the whole, 78 *NcWRKYs* showed a syntenic relationship with those in *P. trichocarpa*, followed by *C. canephora* (74), *A. thaliana* (58), and *O. sativa* (30). The number of orthologous gene pairs between *N. cadamba* and the other species (*P. trichocarpa*, *C. canephora*, *A. thaliana*, and *O. sativa*) was 218, 114, 91, and 45, respectively. More than 90% of the *NcWRKYs* showed a syntenic relationship with *WRKYs* in *P. trichocarpa*, which is higher than *C. canephora* (87%), indicating that *WRKY* genes in *N. cadamba* and *P. trichocarpa* (both as tall arbor) evolved more closely related in evolution. Specifically, *NcWRKY12/38/56* were found to be associated with at least two syntenic gene pairs between *N. cadamba* and *P. trichocarpa,* respectively. *NcWRKY16* and *NcWRKY64* were found to be associated with at least two syntenic gene pairs between *N. cadamba* and *P. trichocarpa*/*A. thaliana*/*O. sativa*, speculating that they may have played an important role in the *WRKY* gene family during evolution. 

### 2.5. Analysis of Promoter Cis-Acting Elements

**Figure 5:**
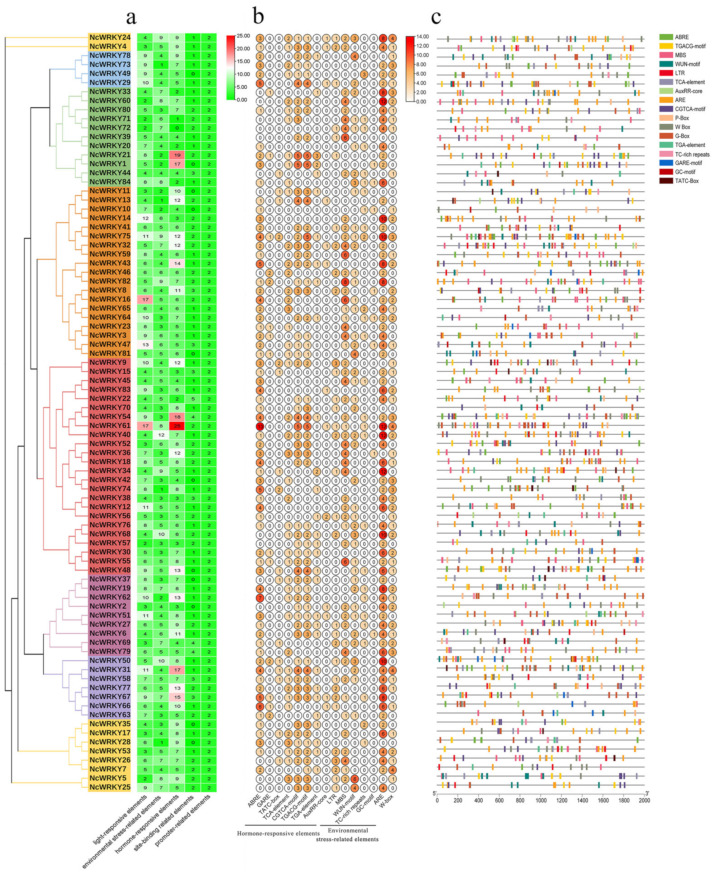
Prediction of cis-acting elements in promoters of *NcWRKYs*. (**a**) The number of five types of cis-acting elements in each *NcWRKYs* promoter. (**b**) The number of different elements in hormone-responsive and environmental-stress-related elements. (**c**) Visualization of environmental-stress-related and hormone-responsive elements, W-box and G-box in *NcWRKYs* promoters.

To further understand transcriptional regulation and the potential functions of *NcWRKYs* in *N. cadamba*, the *cis*-acting elements of *NcWRKY* promoters were predicted using PlantCARE. In addition to the well-characterized site-binding-related elements and promoter-related elements, three categories of cis-regulatory elements were found to be highly concentrated in the promoter region of *NcWRKYs*, including light-responsive, hormone-responsive, and environmental-stress-related elements. The most abundant elements in *NcWRKYs* promoters were hormone-responsive elements, which were represented by eight types (Figure 5a). Specifically, ABRE elements (response to ABA) were the most widely distributed and presented in over half of the identified *NcWRKYs.* Moreover, several environmental-stress-related cis-acting elements responsible for response to low-temperature (LTR), drought (MBS), wound (WUN motif), stress (TC-rich repeats and ARE), and anerobic (GC motif and ARE) were identified (Figure 5b). Specifically, ARE element was prevalent and displayed across most of the *NcWRKY* promoters, implying that *NcWRKYs* were responsive to anerobic stress. Taken together, these results suggested the potential role of *NcWRKYs* in response to stress and hormone signaling pathways. Interestingly, some *NcWRKYs* comprised more than 2 W-box (TTGACC) in their promoters, such as *NcWRKY7*, *NcWRKY24*, *NcWRKY31*, and *NcWRKY61* have four W-box, indicating that these *NcWRKYs* have the potential function to regulate plant’s defensive response to stresses by self-regulating its expression and the cross-talk between different WRKY TFs.

### 2.6. Protein Interaction Network of NcWRKY

**Figure 6:**
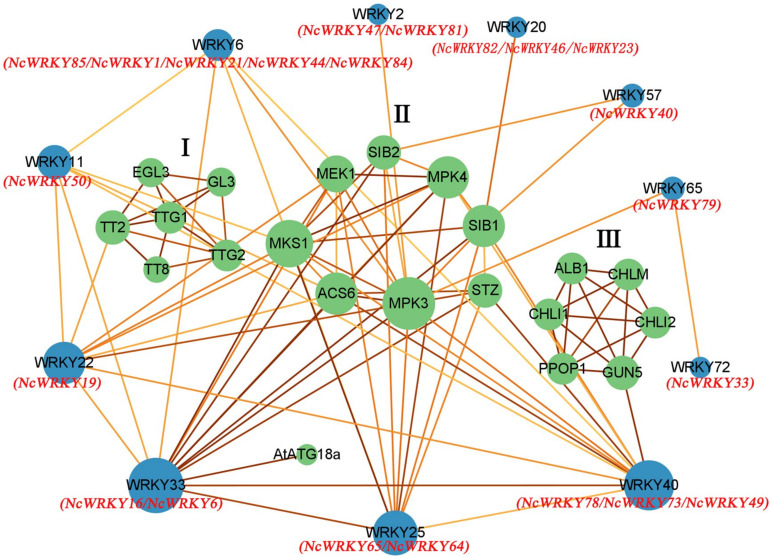
Protein–protein interaction network for NcWRKY proteins based on their orthologs in *A. thaliana*. The green and blue circles represented the interacting proteins found in String and orthologs proteins in *A. thaliana*, respectively. The red name below the blue circle indicates the WRKY protein orthologous to this protein in *N. cadamba*. The abbreviated names were the genes of *A. thaliana*. The color of the line represents the strength of the interaction, in which a darker line indicated higher reliability.

To elucidate the biological functions and regulatory network of NcWRKY proteins, the 18 homologous WRKY proteins in *A. thaliana* were used to predict the protein–protein interaction network of NcWRKY proteins. The results of homologous similarity were shown in Supplementary File S3. The results indicated 11 AtWRKY proteins and 21 corresponding interacting functional proteins which can be divided into three groups (Figure 6). Most AtWRKY proteins interact with more than one protein, and eight proteins can interact with more than two other functional proteins. Some proteins that interact with each other in a group and only interact with one AtWRKY protein. Group 1 (EGL3, GL3, TT2, TT8, TTG1, and TTG2) only interact with AtWRKY22 by TT2 and Group 3 (CHLM, CHLI1, PPOP1, CHLI2, ALB1, and GUN5) only interact with AtWRKY40 by GUN5. According to the biological process analysis in the GO database, proteins of Group 1 were related to epidermal cell fate specification, positive regulation of anthocyanin biosynthetic process, trichome differentiation, and jasmonic-acid-mediated signaling pathway (Supplementary File S4). Proteins of Group 3 were related to the chlorophyll biosynthetic process and aromatic compound biosynthetic process. Proteins of Group 2 were mainly related to MAPK cascade which responds to abiotic and biotic stresses, such as defense and immune system processes, and responds to osmotic and extreme temperature stresses [30,31,32]. This group also included positive regulation of autophagy, cellular heat acclimation, and camalexin biosynthetic process. AtWRKY33 (orthologous with NcWRKY8 and NcWRKY16), an important TF in abiotic stress [12,33] and phytoalexin biosynthesis [34], played a key role in the whole protein interaction network, especially in MAPK cascade. AtWRKY22 (orthologous with NcWRKY19) and AtWRKY25 (orthologous with NcWRKY64 and NcWRKY65) participated in aging and response to cold, respectively.

### 2.7. Expression Patterns of NcWRKYs Gene in N. cadamba Tissues by RNA-Seq

The expression patterns of all 85 *NcWRKYs* in the transcriptome data, which was derived from different tissues, including bud, young leaves, old leaves, bark, cambium, phloem, root, and young fruit, were investigated in this study (Figure S2, Supplementary File S5). The results showed that all 85 NcWRKYs were expressed in different tissues (FPKM > 0), and they were generally highly expressed in roots and old leaves. Some of the *NcWRKYs* were not expressed in the tissues we tested due to special temporal and spatial expression patterns.

**Figure 7:**
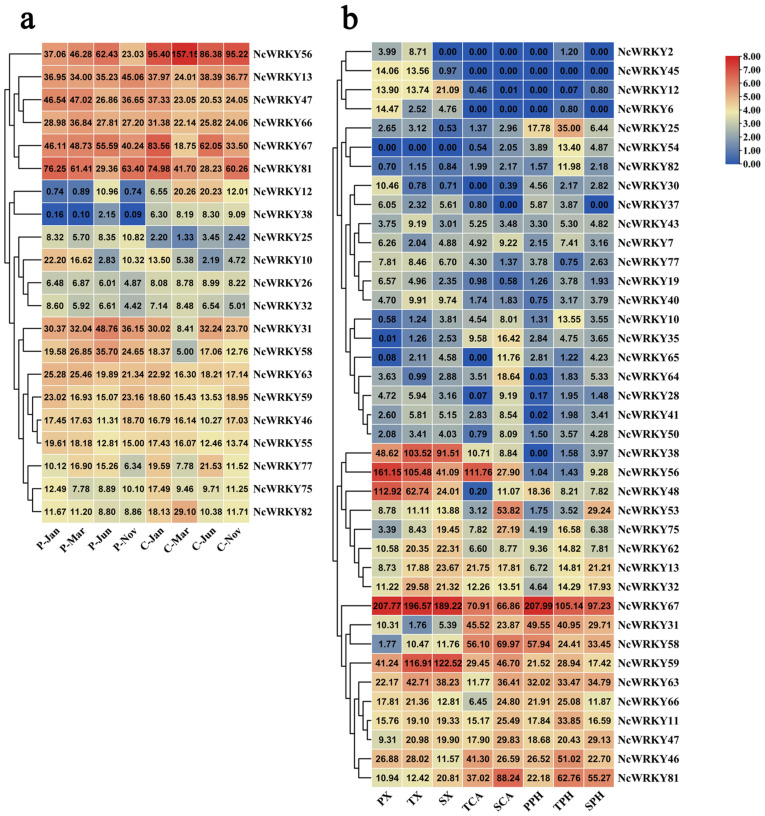
*NcWRKYs* transcriptional levels at different developmental stages of cambium, phloem, and xylem and combined analysis by laser capture microdissection (LCM)-RNA-seq. The heatmaps were created by TBtools based on the transformed data of log2 (FPKM+1) values, and the cluster analysis was performed on gene expression level by row. (**a**) Expression of *WRKY* genes in cambium and phloem in different months. C and P indicated cambium and phloem, respectively, and suffixes ‘Jan, Mar, Jun, and Nov’ indicate January, March, June, and November, respectively. (**b**) Expression of *WRKY* genes in xylem, cambium, and phloem at the stages of primary growth, secondary growth, and transitional stages from primary to secondary growth. PX, primary xylem; TX, xylem at the transitional stage from primary to secondary growth; SX, secondary xylem; TCA, cambium at the transitional stage from primary to secondary growth; SCA, secondary cambium; PPH, primary phloem; TPH, phloem at the transitional stage from primary to secondary growth; SPH, secondary phloem.

Since the wood formation is of great significance for perennial trees, we further focused on the analysis of the expression patterns in different seasonal stages of cambium and phloem. *NcWRKY13/47/56/66/67/81* had high expression in both cambium and phloem in different seasons in the whole year (Figure 7). *NcWRKY12* and *NcWRKY38* had a similar expression mode, with higher expression in cambium but only expressed in phloem of June. To further explore the expression profiles of *NcWRKY* in different developmental vascular tissues, we isolated cambium, phloem, and xylem cells by laser microdissection at three stages, including primary growth, secondary growth, and the transition stage from primary to secondary growth, and the RNA-seq was subsequently carried out (data not published). The results showed that 39 *NcWRKYs* exhibited different expression levels during diverse phases of wood formation, suggesting that these *NcWRKY* genes played distinct roles during wood formation. For example, five *NcWRKYs* (*NcWRKY38/48/56/59/67*) had high expression in xylem of all three phases of wood formation, of which *NcWRKY67* exhibited the highest expression, suggesting that these genes played important roles in regulating xylem development. In cambium, *NcWRKY56*, *NcWRKY67*, and *NcWRKY58* showed the higher expression in the transition stage, while *NcWRKY81* had the highest expression in the stage of secondary growth (Figure 7b). Moreover, some *NcWRKYs* had high expression in three different wood development stages, such as *NcWRKY38/46/66/56/59/63/67*, suggesting that these genes may participate in the development of stem of *N. cadamba*. Altogether, these results indicated that *NcWRKY* genes evolved diverse biological functions that were important for wood formation.

### 2.8. Expression Patterns of NcWRKYs in Response to Different Treatments

**Figure 8:**
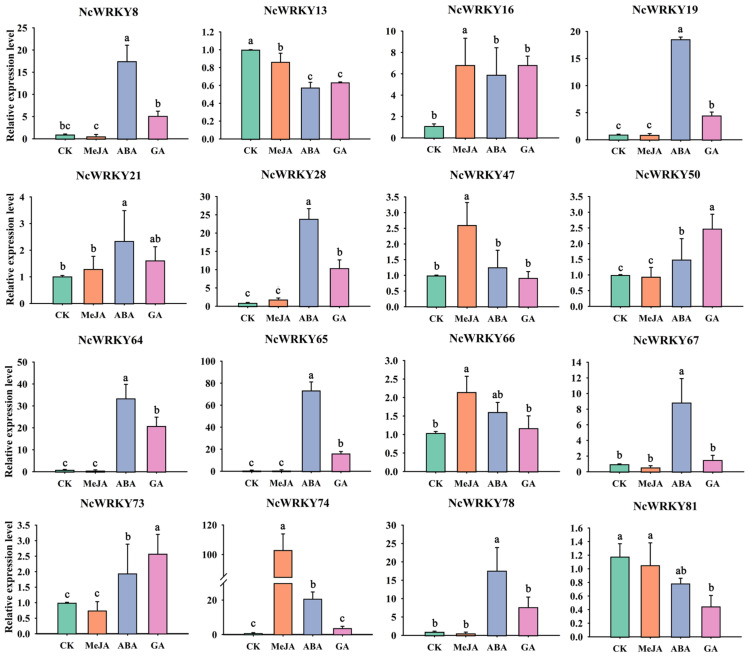
Expression profiles of 16 selected *NcWRKYs* in response to various hormone treatments, including MeJA, ABA, and GA. *NcUPL* was used as the endogenous control. Error bars indicated the standard deviation of three biological replicates, each containing three technical replicates. The same letters indicate groups that were not significantly different from each other according to Duncan’s multiple range test, *p* = 0.05.

To further confirm whether the expression of *NcWRKYs* was influenced by different abiotic stresses and hormonal treatments, RT-qPCR was used to examine the expression patterns of the 16 selected *NcWRKYs* in different treatments, including ABA, MeJA, GA, PEG6000, and NaCl. As to various hormone treatments (Figure 8), most of the 16 *NcWRKYs* responded to multiple treatments at the transcriptional level. For instance, *NcWRKY64* was induced by all tested treatments, except MeJA treatment. Some genes exhibited the same response pattern, such as *NcWRKY28/64/78*, which was strongly induced by ABA and GA, but slightly upregulated under MeJA treatment. On the contrary, *NcWRKY50* and *NcWRKY73* change little in different hormone treatments. In addition, several *NcWRKYs* were simultaneously induced by one treatment. For example, *NcWRKY8/19/28/65/67/74/78* were induced by ABA treatment (the fold change was more than 8), *NcWRKY8/16/28/64/65/78* were induced by GA_3_ treatment (fold change > 5), and five *NcWRKY16/28/47/66/74* were induced by MeJA. Conversely, some genes were down-regulated after treatment, such as the transcript levels of *NcWRKY78* were decreased to half of the control by MeJA treatment, and *NcWRKY13* and *NcWRKY81* were repressed after ABA and GA_3_ treatments. 

**Figure 9:**
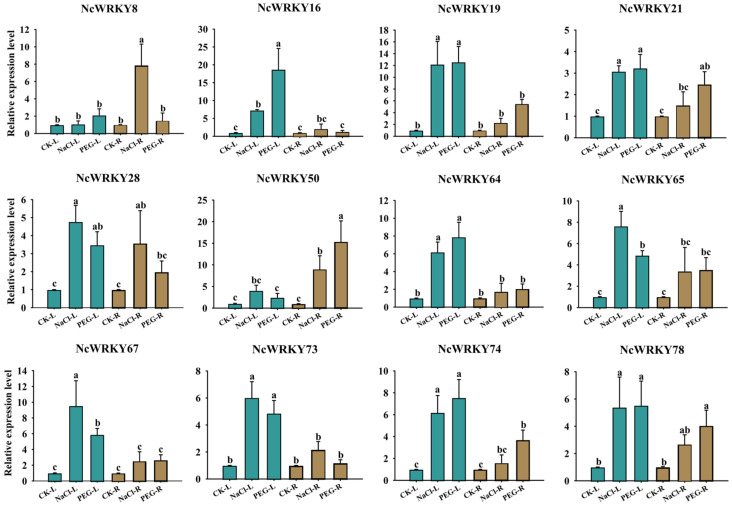
Expression profiles of 12 selected *NcWRKY* genes in response to NaCl and PEG6000 treatments. The leaf and root were collected after NaCl and PEG stress treatments and used for the detection of *NcWRKYs* expression. The suffix ‘L’ and ‘R’ refers to the leaf and root, respectively. *NcUPL* was used as the endogenous control. Error bars indicated the standard deviation of three biological replicates, with each comprising three technical replicates. The same letters indicate groups that were not significantly different from each other according to Duncan’s multiple range test, *p* = 0.05.

For PEG6000 and NaCl treatments, we selected 12 *NcWRKY* genes for detecting their expression by RT-qPCR. As shown in Figure 9, most genes were more strongly responsive in leaves compared to roots, such as *NcWRKY16/19/64/65/73*. On the contrary, *NcWRKY50* had a stronger response intensity in roots. Four of the twelve genes (*NcWRKY21/28/65/78*) had little difference in expression between leaves and roots under PEG6000 and NaCl treatments. Interestingly, *NcWRKY8* only had a comparatively large response intensity in the roots of NaCl treatment.

### 2.9. MeJA Promotes Cadambine Biosynthesis and NcWRKYs Expression

**Figure 10:**
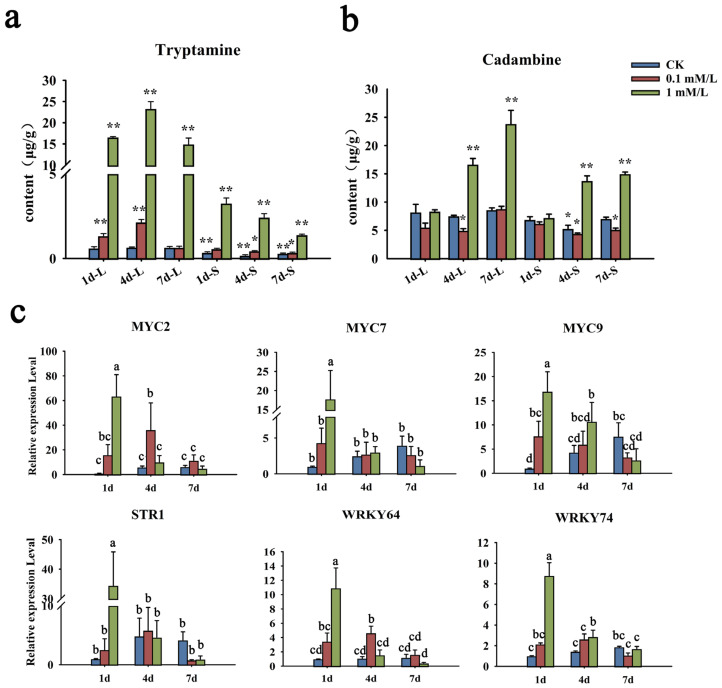
Changes of metabolite content and gene expression after MeJA treatment. (**a**,**b**) The content change of tryptamine and cadambine under MeJA treatment. Quantitative determination of tryptamine and cadambine was performed by UHPLC-MS-MS. (**c**) Transcript levels of *NcMYCs*, *NcSTR1*, and *NcWRKY64/74* in leaves of *N. cadamba* seedlings under MeJA treatments. The expression levels were determined by qRT-PCR. *NcMYCs*, as a kind of key transcriptional factor of MeJA signaling pathway, reflect the response of the jasmonic acid signal pathway after treatment. *NcSTR1* can catalyze the synthesis of strictosidine in *N. cadamba*, reflecting whether MeJA treatment affects the expression of synthesis pathway genes. *NcRPL* was used as the reference gene. Error bars represented the standard deviation of three biological replicates. The same letters indicate groups that were not significantly different from each other according to Duncan’s multiple range test, *p* = 0.05. Statistically significant differences compared with the CK (untreated control) at 1 d were determined by *t*-tests (* *p* < 0.05, ** *p* < 0.01).

MeJA, as an exogenous hormone, can not only play a defensive role in plants, but also widely regulate the synthesis of a variety of secondary metabolites. The *N. cadamba* plants were treated with 0.1 mM/L and 1 mM/L of MeJA, and the contents of cadambine and tryptamine, the precursor of cadambine, were tested by UHPLC-MS-MS (Supplementary File S6). In leaves, the content of tryptamine was obviously elevated at 1 d and reached its highest at 4 d in the 1 mM/L MeJA treatment group. At 7 d after MeJA treatment, it dropped to the same level as 1 d (Figure 10a). However, the accumulation of cadambine gradually increased and reached the highest abundance at 7 d. In the stem, the content of tryptamine was slightly decreased from 1 d to 7 d. On the contrary, cadambine accumulation was obviously elevated at 4 d and slightly enhanced at 7 d (Figure 10b). As for the treatment of 0.1 mM/L, the changes of these two metabolites’ accumulation were similar to that of 1 mM/L, but with gentle enhancement in both leaves and the stem. 

As the key intermediate of the cadambine biosynthetic pathway, *NcSTR1* catalyzed the synthesis of strictosidine in *N. cadamba* [29]. Moreover, MYC is inhibited by JAZ protein, which leads to the downstream regulatory switch being turned off. When MeJA acted as an activation signal, JAZ protein would be degraded, and then the MYC transcription factor in the inhibited state was released, which thereby activates the target gene expression activity [35,36]. Accordingly, we further detected the expression patterns of *NcMYCs*, *NcSTR1* (evm. model. Contig 69.90), and *NcWRKYs* after MeJA treatment by RT-qPCR (Figure 10c). For the 1 mM/L MeJA treatment group, three *NcMYCs*’ (*NcMYC2*, *7,* and *9)* expression was strongly induced by MeJA, with a peak after 1 d, and gradually decreased after 4 d and 7 d. The expression of the *STR1* gene and two *WRKY* genes (*NcWRKY64* and *NcWRKY74*) was similar to *NcMYCs*, with the same pattern. However, *NcMYC2*, *STR1*, *NcARKY64*, and *NcWRKY74* were induced at later time points (i.e., 4 d) in the 0.1 mM/L MeJA treatment group, suggesting that a higher concentration of MeJA was essential for rapid induction of expression of these genes. A control time course of treatment with the solvent ethyl alcohol showed no effect on the expression of *NcARKY64* and *NcWRKY74*, but promotes the expression of *NcMYC* and *STR1* genes. More *NcWRKYs* were upregulated significantly after treatment with 1 Mm/L MeJA (Figure S3).

## 3. Discussion

The WRKY family is a large transcription factor family and plays pivotal roles in higher plants. Identification and characterization of *WRKY* genes have been performed in many plants, including the model plants, crops, and medicinal plants, such as Arabidopsis [3], corn [37], tomato [38], ginseng [39], and poplar [40]. In the present study, a total of 85 *NcWRKY* genes were identified in the genome of *N. cadamba* by bioinformatics analysis. Their features, expression patterns, and putative function were studied in detail. 

### 3.1. Diverse Characterization of WRKY in N. cadamba

The uneven gene family size of WRKY was found in different plants. A previous report had indicated that the WRKY members ranged from 59 to 161 among seven species, of which *Z. mays* had the most prominent WRKY family members (genome size 2100 Mb), and a minor WRKY family was found in *V. vinifera* (genome size 427 Mb) [39]. *N. cadamba* had moderate WRKY family members (85), in accordance with the moderate genome size (744.5 Mb). These results indicated that the number of WRKY family members was correlated with genome size. However, an exception was found in ginseng, in which its genome size (2900 Mb) is larger than that of *Z. mays*, but with fewer WRKY family members [39]. Interestingly, *N. cadamba* possesses comparatively a greater number of *WRKY* genes compared with many dicotyledonous plants, including *Solanum lycopersicum* (81 *WRKY* genes), *A. thaliana* (72 *WRKY* genes), *D. carota* (67 *WRKY* genes), *Salvia miltiorrhiza* (61 *WRKY* genes), and *C. roseus* (47 *WRKY* genes) [41]. Gene duplication was found to play a very important role in the expansion of the *WRKY* gene family. In *N. cadamba*, a total of 76 segmental duplication events are identified in *NcWRKYs*, and the genes involved in segment duplication do not have distinct subgroup distributions, indicating that the WRKY family has no obvious evolutionary differences in the distribution of *N. cadamba*. Moreover, for all pairs, the Ka/Ks ratios are <1, indicating that the *WRKY* gene family in *N. cadamba* has undergone purifying selection rather than positive selection, and indicating that the *NcWRKYs* are highly conserved.

The WRKY domain is the key sequence that determines the specific binding of the WRKY protein to the *cis*-element W-box. According to previous studies, variations of the WRKYGQK motif in the WRKY domain might influence normal interactions of WRKY genes with downstream target genes [42]. The multiple sequence alignment results revealed that six NcWRKY proteins (NcWRKY30/48/55/57/68/76) in Group IIc had sequence variation in their WRKY domain (WRKYGKK), which binds specifically to the WK-box (TTTTCCAC) in tobacco [43]. According to transcriptome data, these six *NcWRKYs* had similar expression patterns, mainly expressed in old leaf, young fruit, and root (Additional Figure 2). The result indicated that the variation of the WRKY domain in *N. cadamba* may not affect its binding function, and the six *NcWRKYs* may have similar functions. It might be worth to further investigate whether the mutated domain confers a special function. The diversification of exon/intron patterns played a vital role in the evolution process of many gene families. We found that 85 *NcWRKYs* contained between one and six introns, consistent with that in pineapple [44], cucumber [45], and populus [46]. NcWRKY proteins from Group I have two WRKY domains, and no domain loss events were found in other NcWRKY proteins, which was similar to most of the dicotyledonous plants.

### 3.2. The Potential Function of NcWRKYs in Vascular Development and Response to Hormone and Abiotic Stress

Based on transcriptome data, some valuable clues about the functional role of *NcWRKYs* which are involved in the specific physiological process in *N. cadamba* were obtained. For different developmental stages of cambium, phloem, and xylem, some *WRKY* genes have high expression levels, and these genes were highly overlapping, such as *NcWRKY12/38/56* specifically expressed in different developmental stages of cambium and phloem. These genes were orthologous with the *MiWRKY12* and *PtrWRKY19*, which participate in pith secondary cell wall formation in *Miscanthus* and *P. trichocarpa*, respectively [47,48]. Moreover, in *atwrky13* mutants, lignin-synthesis-related genes were repressed, and the number of sclerenchyma cells, stem diameter, and the number of vascular bundles were reduced [49]. *NcWRKY12/38/56* were in the same subgroup as *AtWRKY13,* suggesting that they might involve in the stem development of *N. cadamba.* Accordingly, we inferred that these three *NcWRKYs* may participate in vascular development. 

A variety of conserved cis-regulatory elements were shown in the promoter region of *NcWRKYs*, which were involved in a variety of functions, including hormone and abiotic stress responses (Figure 5). The response of *NcWRKYs* to hormone and abiotic stresses can provide valuable clues to reveal the potential role of *WRKY* genes in *N. cadamba*. In this study, 16 *NcWRKYs* chosen from different groups were subjected to hormones, salt, and drought stress treatments profiled by means of RT-qPCR. The result shows that all of *NcWRKYs* can be induced by at least two hormones, implying that a single *WRKY* gene can be regulated by various hormones, similar to that in Arabidopsis, *AtWRKY18/40/60* were shown to participate in signaling pathways that were mediated by plant hormones SA, JA, and ABA [8], and Group III members of *CsWRKYs* in *Cymbidium sinense* were strongly induced in response to various hormone treatments [50]. Previous studies have revealed that WRKYs are important regulators in linking hormone signaling in response to environmental stresses [51,52]. *FcWRKY40* from *Fortunella crassifolia* was involved in ABA signaling pathways and positively regulated salt tolerance by directly binding to and activating the promoters of *FcSOS2* and *FcP5CS1*. The transcriptions of *NcWRKY8/16/64*/*65* were promoted simultaneously by ABA and drought stress. In addition, several stress-related transcriptional regulatory elements, including an ABA-responsive element and MYB binding site involved in drought responses, were found in these NcWRKYs. These results suggested that these NcWRKYs may regulate drought responses through ABA-dependent signaling pathways, but this remains to be elucidated in further study. Moreover, these NcWRKYs have a phylogenetically closest relationship with *AtWRKY33*, a typical ABA and drought-responsive *WRKY* gene in *A. thaliana* [53,54], supporting that they play important roles in ABA signaling pathway and response to drought stress.

### 3.3. The Involvement of NcWRKYs in Cadambine Biosynthesis

Jasmonic acid (JA) is an important elicitor in plant secondary metabolism at the transcriptional level by altering the expression of a set of biosynthesis genes [55,56]. It has been shown that methyl-jasmonate (MeJA) induced the expression of the terpenoid indole alkaloid (TIA) biosynthesis genes, including the strictosidine synthase (STR) gene [55], resulting in promoting TIA metabolism in *Catharanthus roseus* [57]. In this study, cadambine and the key intermediate product tryptamine were both induced under MeJA treatment, suggesting that MeJA acted as an elicitor in cadambine biosynthesis. Our results facilitated that MeJA plays a significant role in plant metabolism. In addition, the MYC gene family is a kind of key TF in the MeJA signal pathway [58]. The expression levels of *NcMYCs* were highly induced after MeJA treatment (Figure 10c), indicating that the MeJA signal pathway was activated in *N. cadamba* seedlings.

Studies have indicated that WRKY proteins participated in transcriptional regulating biosynthesis of secondary metabolites, including alkaloids [24], volatile terpenes [59], and anthocyanin [60]. It has been reported that several WRKY proteins may regulate secondary metabolism biosynthesis in response to JA elicitation. In cotton, GaWRKY1 was strongly induced by MeJA and participated in the regulation of sesquiterpene phytoalexin biosynthesis by transactivating the promoter of the (1)-δ-cadinene synthase (CAD1) gene [20]. The expression pattern of *NcWRKY64* and *NcWRKY74* was consistent with that of *NcMYCs* and *NcSTR1*, which were upregulated after MeJA treatment. In addition, there were two WRKY TF binding site W-box (TTGACC) within 2000 bp upstream of the NcSTR1 promoter (Figure S4). We proposed that NcWRKY64/74 has the potential function of positively regulating the biosynthesis of cadambine by activating NcSTR1 in response to MeJA. The interaction between NcWRKY64/74 and NcSTR1 remained to be investigated in the future study. 

Furthermore, AtWRKY33, OpWRKY6, CrWRKY1, and TcWRKYs were involved in the regulation of camalexin (Indole alkaloids), camptothecin (Quinoline alkaloid), vinblastine (Indole alkaloids), and taxol (Diterpene alkaloids) biosynthesis, respectively [18,23,24,61,62]. AtWRKY33 and NcWRKY64, OpWRKY6 and NcWRKY74 were close to each other in the phylogenetic tree (Figure S5), and they have the same WRKY domain (WRKYGQK) and zinc-finger motif (C-H_4_-X_23_-H-X-H) (Figure S6). Therefore, according to the high homology between AtWRKY33, NcWRKY64, and NcWRKY74, we inferred that NcWRKY64 and NcWRKY74 were essential for MeJA-responsive TIA accumulation in *N. cadambia*. No MeJA-responsive cis-acting element was found in the *NcWRKY74* promoter, suggesting that the expression of *NcWRKY74* was induced by MeJA in an indirect manner. Further functional characterization of *NcWRKY64* and *NcWRKY74* by overexpression or knockout of these *NcWRKY* genes may help to elucidate their function in TIA biosynthesis, which will provide crucial information for understanding the MeJA-WRKY-STR regulatory module in the cadambine biosynthesis. 

## 4. Materials and Methods

### 4.1. Data Sources

All raw and processed sequencing data used in this study were derived from the previous research of our research group and can acquire in the NCBI BioProject database under accession number PRJNA650253. The raw sequencing data of the resequencing data and transcriptome were downloaded from the NCBI BioSample database under accession numbers SAMN15700860 and SAMN15700859, respectively. The genome annotation and assembled genome sequences were from the Figshare website (https://Figshare.com/s/ed20e0e82a4e7474396b) [29]. The AtWRKY protein sequences were downloaded from the *A. thaliana* Information Resource website (https://www.arabidopsis.org/index.jsp).

### 4.2. Identification of WRKY Genes in N. cadamba

To identify all candidate WRKY genes in *N. cadamba*, a BLASTP search with a threshold e-value of 1 × 10^−5^ was performed using *A. thaliana* WRKY protein sequences as query sequences. The hidden Markov model (HMM) file corresponding to the WRKY domain (PF03106) was downloaded from the Pfam database (ftp://ftp.ebi.ac.uk/pub/databases/Pfam/current_release/Pfam-A.hmm.gz). The Simple HMM Search program of TBtools [63] was used to search all the potential WRKY-domain-containing protein sequences in the *N. cadamba* genome. The unique potential WRKY proteins of *N. cadamba* based on the results of BLASTP and Simple HMM Search were further validated using the SMART (http://smart.embl.de/smart/batch.pl) and conserved domain database from NCBI (https://www.ncbi.nlm.nih.gov/cdd/) to determine that they indeed contained the core domain sequences. Eighty-five NcWRKY proteins in *N. cadamba* were finally obtained. The online tools from ExPASy (https://web.expasy.org/protparam/) were used to analyze the amino acid number, isoelectric point (pI), and MW of NcWRKY proteins. Subcellular localizations were predicted by the Plant-mPLoc website. (http://www.csbio.sjtu.edu.cn/bioinf/plant-multi/). 

### 4.3. Phylogenetic Analysis of WRKY Family Members and Sequence Alignment

A phylogenetic tree with 72 AtWRKY protein sequences and the identified NcWRKY proteins sequences was constructed by One Step Bulid, an ML Tree program from TBtools [63] with default parameters, followed by visualization and optimization in iTOL [64]. The WRKY family members from *A. thaliana* were used as a reference for the classification of the WRKY family members in *N. cadamba*. All WRKY domain sequences of candidate NcWRKY proteins were aligned using DNAMAN software.

### 4.4. Analysis of Conserved Motifs, Conserved Domains, and Gene Structures

The conserved motifs of the NcWRKY proteins were analyzed using Simple MEME Wrapper from TBtools [63] with the following parameters: Num of Motifs was 10, Min Motif Width was 6, Max Motif Width was 50, and Max E-value was 10. The conserved domains of NcWRKY proteins were searched by CDD (https://www.ncbi.nlm.nih.gov/cdd/). The conserved motifs, conserved domains, and gene structures which were obtained from the gene structure annotation file were visualized by TBtools. 

### 4.5. Promoter Cis-Regulatory Element Analysis and Chromosomal Localization

The 2000 bp gene sequence upstream of the initiation codon (ATG) of *NcWRKYs* was considered as the gene promoter sequence and deprived of the *N. cadamba* genome by TBtools [63]. *Cis*-acting elements in the promoter region were analyzed using the online software PlantCARE [65] and subsequently visualized using TBtools [63]. Location Visualize from GTF/GFF of TBtools was used to determine the chromosomal position of the identified *NcWRKYs*.

### 4.6. Colinear Analysis and Selective Pressure

To identify the pattern of gene duplication, One Step MCScanX from TBtools [63] with default parameters (E-value cut-off < 1 × 10^−10^ and Num of BlastHits with 5) was used to analyze *WRKY* genes in *N. cadamba* vs. itself and *N. cadamba* vs. *A. thaliana*/*Coffea canephora*/*Populus trichocarpa*/rice, respectively. The results were visualized using TBtools. To assess the selection pressure of genes encoding WRKY proteins, the ratio of nonsynonymous (Ka)/synonymous (Ks) (Ka/Ks is an indicator of selective pressure) was used to evaluate its evolutionary pressure. The values of Ka, Ks, and Ka/Ks were calculated by Simple KaKs Calculator in TBtools [63].

### 4.7. Homology Analyses and Protein Interaction Network Analysis

In order to obtain the orthologous gene pairs and paralogous gene pairs, all the individual NcWRKYs protein sequences were compared against AtWRKY protein sequences using BLASTP (https://blast.ncbi.nlm.nih.gov/Blast.cgi) with the following settings: Total Score >200, Query Cover >than 60%, E-value cut-off < 1 × 10^−10^, and Identity >45%. Using homolog *A. thaliana* WRKYs as a template, the WRKY protein interaction network of *N. cadamba* was analyzed using STRING (https://string-db.org/) with a threshold required score >0.4. The interaction network was further visualized and analyzed using the software Cytoscape 3.9.1.

### 4.8. Plant Material and Stress Treatments

For salinity and drought treatments, 7-week-old clonal plants of *N. cadamba* with uniform height were transferred into MS liquid medium supplied with 100 mM/L NaCl and 10% PEG6000 solution, respectively. The leaves and roots were collected at 8 h. For phytohormone analysis, the seedlings that were transplanted outdoors and grow up to 60 cm after 3 months were respectively sprayed 10 mg/L ABA, GA_3_, and MeJA 50 mL. Then wrap the leaves in fresh-keeping bags. The young leaves were collected 2 d after treatments. All the collected samples were immediately frozen in liquid nitrogen and stored at −80 °C for subsequent analysis.

The 4-month-old *N. cadamba* plants (about 100 cm in height) were sprayed with different MeJA concentrations (0.1 and 1 mM/L) that were mixed with pure water. The mother liquor is 1 mM/mL, which is prepared from MeJA stock solution and absolute ethanol. At about 8:30 every morning, the MeJA was sprayed on the leaf’s front and back surface till dripping for 7 consecutive days. The roots, stems, and leaves of the plants were sampled and stored at −80 °C on the first (1 d), the fourth (4 d), and the seventh (7 d) days after spraying was stopped. Each sample contained three biological replicates. 

### 4.9. Expression Patterns of NcWRKYs

The transcriptome sequencing data of young leaves, old leaves, bud, bark, phloem, cambium, young fruit, and root from 5-year-old *N. cadamba* were collected for RNA extraction for RNA-seq in our previous study [29]. The vascular cells, including cambium, phloem, and xylem cells, at three developmental stages (primary growth, secondary growth, and the transition from primary to secondary growth) were captured by laser microdissection and used for subsequent RNA-seq [66]. The transcript abundance of *N. cadamba WRKY* genes was calculated as fragments per kilobase of exon model per million mapped reads (FPKM). The HeatMap program in TBtools was used to visualize the expression of the target gene obtained from the transcriptome. The HeatMap program in TBtools was used to visualize the expression of the target gene obtained from the transcriptome. Total RNA was isolated from each sample using the E.Z.N.A^®^Plant RNA Kit (Omega, GA, USA). RNA integrity was evaluated by 1% (*v*/*v*) agarose gel electrophoresis, and RNA purity was quantified using an IMPLEN NanoPhotometer. Total RNA was used to synthesize cDNA by using HiScript Ⅲ RT SuperMix for qPCR (+gDNA wiper) (Vazyme, Nanjing, China). The quantitative RT-qPCR was carried out with the Roche Lightcyler^®^ 480 instrument using SYBR Green chemistry. The housekeeping *UPL* and *RPL* genes were used as an internal control of different treatments and MeJA treatment with different concentrations, respectively [67,68]. The RT-qPCR program was set as follows: 95 °C for 30 s, followed by 40 cycles of 95 °C/10 s, 60 °C/30 s, and the fluorescence signal was then read. Subsequently, the procedure was conducted as follows: 95 °C for 15 s, 60 °C for 60 s, and warming up to 95 °C then 50 °C for 30 s; after the fluorescence signal was read, the dissolution curve was analyzed. There were three biological replicates for each sample, and each biological replicate had three technical repetitions. Sequences of the primers used in this study were shown in detail in Supplementary File S7. The data obtained were visualized using SigmaPlot.

### 4.10. Extraction and Quantitative Determination of Tryptamine and Cadambine

The samples were ground at −80 °C using liquid nitrogen. We weighed 0.12 g of sample and added 5 mL of 70% ethanol, and the mixture was held overnight at −20 °C, followed by ultrasonic crushing for 30 min, vibrating every 5 min. After ultrasonic crushing, and centrifugation at 4 °C for 10 min at 7000 rpm, samples were filtered through 0.22 μm membrane filters before liquid chromatography–mass spectrometry (LC-MS) analysis.

Samples were analyzed by reversed-phase chromatography on an Agilent 1290 HPLC, using a 3.0 × 50 mm ECLIPS PLUS C18 column. Water with 0.1% formic acid (A) and methanol (B) was used as the mobile phase component at a flow rate of 0.3 mL/min with the following gradient: 0–3 min, 50% B; 3–6 min, 90% B; 5–6 min, 90% B; 6–10 min, 10% B. A coupled Agilent 6470 MS-QQQ mass spectrometer with ESI and Agilent Jet Stream was used to collect MS data in positive ion mode (parameters: Gas: 300 °C, 8 L/min; Nebulizer: 45 psi; Sheath Gas: 350 °C, 10 L/min; Capillary: 4000 V; VCharging: 1000). Scan type was MRM.

### 4.11. Statistical Analysis

Data from three biological and three technical replicates were used for statistical analysis. All data were indicated by an average of three biological replicate measurements and standard deviation. As for RT-qPCR, the relative gene expression levels were analyzed using the 2^−ΔΔCt^ method. Significance was determined by pairwise comparison using *t*-tests or multiple comparison using Duncan’s multiple range test in SPSS software 26 (*p* = 0.05).

## 5. Conclusions

Overall, a total of 85 *WRKY* genes were identified from *N. cadamba* and divided into seven subfamilies according to their phylogenetic relationships, and members of the same subfamily had similar gene structures and conserved motifs and domains. The analyses of expression patterns based on RNA-seq data revealed their probable functions in different tissues and vascular development. In addition, the detection of remarkable expression profiles of NcWRKYs under hormones and abiotic stresses will provide clues for exploring the signaling pathways in *N. cadambia* in response to hormone and abiotic stresses. It is worth noting that MeJA promoted cadambine accumulation and induced the expression of the key intermediate gene *STR1*, *NcWRKY64*, and *NcWRKY74*, implying the MeJA-WRKY-STR regulatory module in the cadambine biosynthesis. This study suggests a basis for further functional research of the regulatory mechanism of *NcWRKYs* in hormone and stress responses and provides promising candidate genes for regulating cadambine synthesis in *N. cadamba*. 

## Data Availability

Data are provided within the article and Supplementary Materials.

## Supplementary Materials

The following supporting information can be downloaded at https://www.mdpi.com/article/10.3390/ijms24087537/s1.
