# Supplementary material for: Genome-Wide Identification and Expression Analysis of WRKY Gene Family in Neolamarckia cadamba
Source: Int J Mol Sci. 2023 Apr 19;24(8):7537. doi: 10.3390/ijms24087537 (PMC10142840; doi:10.3390/ijms24087537)
Supplement: Supplementary file 1 [file ijms-24-07537-s001.zip › Supplementary Figures.doc]

**Figure S1** Sequence alignment of WRKY domain of 85 NcWRKY proteins. WRKYGQK motif was underlined as WRKY domain, and C2H(H/C) of zinc-finger motif was designated by star (*). NcWRKY23 had incomplete zinc-fingers motif in N-terminal so that its HxH residues were not found. All NcWRKY proteins were grouped according to phylogenetic analysis. The names with N or C represented for N- and C-terminal WRKY domain of Group I respectively.

**Figure S2** The heatmap of *NcWRKYs* in different tissues and processed with log scale normalization. Red represents high expression level and blue represents low expression level.

**Figure S3** Transcript levels of 9 *NcWRKY* genes in *N. cadamba* seedlings under MeJA treatments. The four-month seedlings were treated with MeJA for 7 consecutive days, and leaves were subsequently collected at 1, 4 and 7 d after stopping treatment. The expression levels of *NcWRKYs* were determined by qRT-PCR. *NcRPL* was used as the reference gene. Error bars represented standard deviation of three biological replicates. The same letters indicate groups that were not significantly different from each other according to Duncan’s multiple range test, *p*=0.05.

**Figure S4** The location of two W-box elements in the NcSTR1 promoter.

**Figure S5** Unrooted phylogenetic tree representing relationships among WRKY proteins of *N. cadamba* with CrWRKY1, OpWRKY6, AtWRKY33 and TcWRKYs. A Maximum Likelihood (ML) phylogenetic tree was constructed with 5000 bootstrap replicates.

**Figure S6** Sequence alignment of NcWRKY74/64, OpWRKY6 and AtWRKY33.
